# Animal Venoms—Curse or Cure?

**DOI:** 10.3390/biomedicines9040413

**Published:** 2021-04-12

**Authors:** Volker Herzig

**Affiliations:** 1 GeneCology Research Centre, University of the Sunshine Coast, Sippy Downs, QLD 4556, Australia; vherzig@usc.edu.au; Tel.: +61-7-5456-5382; 2 School of Science, Technology and Engineering, University of the Sunshine Coast, Sippy Downs, QLD 4556, Australia

**Keywords:** venom, toxin, toxicity, lethality, envenomation, antivenom, venoms to drugs, therapeutics, biopesticide, anti-parasitic

## Abstract

An estimated 15% of animals are venomous, with representatives spread across the majority of animal lineages. Animals use venoms for various purposes, such as prey capture and predator deterrence. Humans have always been fascinated by venomous animals in a Janus-faced way. On the one hand, humans have a deeply rooted fear of venomous animals. This is boosted by their largely negative image in public media and the fact that snakes alone cause an annual global death toll in the hundreds of thousands, with even more people being left disabled or disfigured. Consequently, snake envenomation has recently been reclassified by the World Health Organization as a neglected tropical disease. On the other hand, there has been a growth in recent decades in the global scene of enthusiasts keeping venomous snakes, spiders, scorpions, and centipedes in captivity as pets. Recent scientific research has focussed on utilising animal venoms and toxins for the benefit of humanity in the form of molecular research tools, novel diagnostics and therapeutics, biopesticides, or anti-parasitic treatments. Continued research into developing efficient and safe antivenoms and promising discoveries of beneficial effects of animal toxins is further tipping the scales in favour of the “cure” rather than the “curse” prospect of venoms.

## 1. Introduction to Venomous Animals and Their Venoms

**Figure 1:**
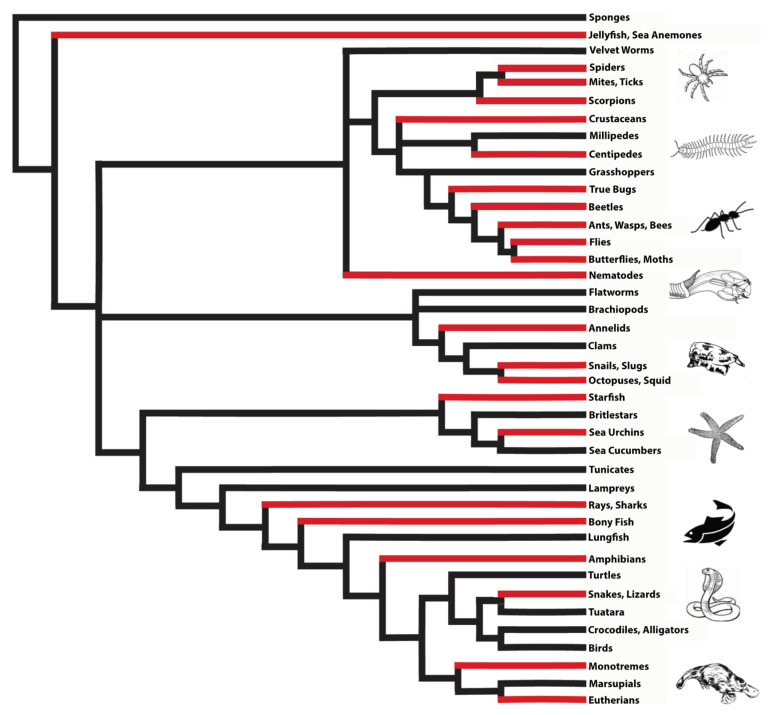
Evolutionary tree of animals (modified from [1]). Lineages with venomous representatives are indicated in red.

With an estimated 220,000 species or 15% of the global animal biodiversity being venomous [1], the majority of all animal lineages (57.5%) actually contain venomous representatives (Figure 1). Venom usage has convergently evolved in many animal lineages, and a recent estimate for arthropods, which are by far the most speciose venomous animals, suggested that venom systems have independently evolved in at least 19 lineages or even 29 times, if secretions that facilitate hemolymph or blood-feeding parasitism are also accounted for [2,3,4]. In comparison, flight (which, like venom, is also a trait that can endow animals with an evolutionary advantage but that comes at a high energetic cost) has convergently evolved only four times in the animal kingdom (i.e., in birds, bats, insects, and pterosaurs) [5]. Thus, with the exceptional abundance of independent evolutionary origins of animal venoms comes a vast diversity of venom system anatomies and venom application strategies. Direct injection into prey or predators has been realised via modified fang-like extremities (spiders, centipedes, crustaceans), antennae (beetles), pincers (pseudoscorpions), modified teeth (snakes), beaks (octopuses), stingers (scorpions), modified ovipositors (hymenopterans), proboscis (flies and bugs), barbs (fish), spurs (monotremes), hairs (caterpillars), harpoons (cone snails), and nematocysts (jellyfish, sea anemones). Even external application by spraying (snakes, scorpions, ants) [6,7,8] or release of toxins into the surrounding aqueous environment (cone snails) have also been reported [9]. Poisonous (i.e., lacking a morphological structure for direct venom delivery) amphibians have glands for the secretion of their toxins in order to deter predators when being ingested [10]. However, while the delivery strategies might differ between poisonous and venomous animals, the main purpose of animal poisons and venoms is to cause physiological changes that incapacitate or deter the targeted victim, primarily for predatory or defensive reasons. These physiological changes can affect a variety of molecular targets and form the basis of both the detrimental as well as beneficial aspects of these toxic secretions. For this reason, this Editorial and the related Special Issue of *Biomedicines* cover both perspectives in relation to animal poisons, venoms, and toxins.

## 2. History of Human Interactions with Venomous Animals

Humans have always been fascinated by venomous and poisonous animals, with their toxic secretions being exploited for traditional medicine for thousands of years. The usage of honeybee venom for a variety of therapeutic applications [11] dates back to at least the second century BC in Eastern Asia [12,13]. Leeches have also been used by many ancient cultures (e.g., Egyptian, Indian, Greek, and Arabian), mainly for bloodletting, but also for the treatment of diseases such as inflammation, skin diseases, rheumatic pain, or reproductive problems [14]. Various Amazonian tribes are known to utilise painful ant stings for their puberty rituals [15]. Another interesting example is poison dart frogs from the family Dendrobatidae, which have been employed by several South American tribes as a source of poison to cover their arrow tips used for hunting [16]. It is a remarkable twist that poison dart frogs do not even produce their toxic alkaloids themselves, but sequester them (in the majority of cases in an unmodified form) from their mostly arthropod diet (e.g., ants and millipedes) [17]. Thus, the human usage of poison dart frog alkaloids can be considered a sequential recycling of toxic compounds originating from arthropods. Furthermore, this example might also blur the separation between venoms and poisons, if venom components are recycled into a poisonous secretion. Some venomous animals such as honeybees have even been domesticated, with records of beekeeping dating back at least 4500 years to Egypt [18] or 3000 years to Israel [19]. In modern agriculture, honeybees are indispensable as production animals for the pollination of a wide variety of crops to ensure the survival of billions of people, with the yield of honey and other bee products merely being an added economic benefit. In the last few decades, a number of other venomous animals have even made the status of human “pets”, with a growing global scene of enthusiasts mainly in developed countries keeping venomous snakes, spiders, scorpions, insects, and centipedes in their homes [20].

## 3. The “Curse”: Detrimental Effects of Animal Venoms

In its infancy, venom research was incited by an urgent need for antivenoms to combat human fatalities caused by envenomations from snakes, spiders, and scorpions [21,22]. The most common strategy for developing antivenoms comprises injecting small and then increasing doses of venoms into mammals (e.g., horses, sheep, or rabbits) and then isolating the antibodies produced in their blood as antivenom for treating envenomated humans. The production of heterologous antivenoms was pioneered by Albert Calmette in 1895 to raise cobra antivenom [22] and has since been successfully adapted to a range of other venomous animals. Nowadays, antivenoms are available against a wide range of venomous animals including spiders (*Phoneutria*, *Loxosceles*, *Atrax*, *Latrodectus*), scorpions (only from the family Buthidae, e.g., the genera *Androctonus, Buthus, Centruroides, Leiurus, Parabuthus,* and *Tityus*), ticks (*Ixodes holocyclus* = “paralysis tick”), caterpillars (*Lonomia obliqua*), box jellyfish (*Chironex fleckeri*), stonefish (*Synanceia*), and many species of snake (belonging to the families Elapidae and Viperidae). Modern molecular techniques have also started to tackle a major disadvantage of heterologously produced antivenoms, which is their potential incompatibility with the human immune system. A recent study, for example, showed that oligoclonal mixtures of recombinant human immunoglobulin G can be successfully and cost-efficiently used for neutralising snake venoms [23].

The majority of commercially available antivenoms are already targeted against snakes. Nevertheless, a large deficit still remains in developing effective antivenoms for treating snake envenomations. The reason that snakes have to be considered as the most dangerous venomous animals from a human perspective is not only their large venom amounts but also the fact that most snakes have evolved their venoms to overcome vertebrate prey and therefore many of their toxins also exhibit activity in humans. Another reason is that some snake antivenoms lack cross-reactivity and are therefore only effective to treat envenomations from the particular (or closely related) snake species against which they were raised [24]. Thus, even the use of polyvalent (i.e., raised against several species) antivenoms will be limited to certain geographical areas and cannot simply be applied on a larger or even global scale [24]. Unfortunately, the countries that are most affected by snake envenomations are usually those that are most economically disadvantaged and therefore lack the funding and expertise required for the development of snake antivenoms specific to their region [25]. This became particularly obvious when the commercial production of Fav-Afrique was discontinued in 2014 for economic reasons, which is estimated to have resulted in an additional 10,000 annual deaths in Africa [26]. Due to the global scale of 1.8–5 million annual snake envenomations, the resulting 81,000–138,000 fatalities, and the even larger number of permanent disfigurements and disabilities, snake envenomations have been reclassified as a neglected tropical disease by the World Health Organisation [24,25,26]. Additionally, due to poor record keeping and many unreported cases in developing countries affected by snake envenomations, these staggering numbers might even be a gross underestimate of the real numbers [24]. While a range of other venomous organisms, including arachnids, hymenopterans, cone snails, and jellyfish, have also been reported to cause human fatalities [27,28,29,30], their resulting global fatality numbers are dwarfed by the number of snakebite fatalities. Nevertheless, some of these animals, such as scorpions, can be responsible for a large number of fatalities in those geographical regions where they occur in high population densities [27,31,32,33].

## 4. The “Cure”: Beneficial Effects of Animal Venoms

In recent decades, the majority of toxinologists have shifted their attention towards potential benefits of toxins from animal venoms for humanity, with various applications, including diagnostics [34], therapeutics [35,36,37,38,39,40,41,42], molecular tools in basic research for studying physiological processes [43,44,45,46,47], and treatments against pests and parasites [48,49]. Most of these applications involve peptide toxins and rely on their exquisite potency and selectivity against their respective molecular targets [50], but also their stability [51] and economical means of production at a large scale [52]. In cases where a molecular target of an animal toxin is also involved in the pathophysiology of a disease, this can then be exploited to develop novel therapeutics [39]. So far, six venom-derived drugs have made it to the market, including an antidiabetic peptide from a lizard, an analgesic peptide and a monomeric insulin from cone snails, a sea anemone peptide for treatment of autoimmune disease, a scorpion peptide for imaging brain tumours during surgery, and a spider peptide bioinsecticide (for further details, see [36]). Moreover, many more animal toxins or toxin-derived drugs are still in the pipeline for a wide variety of potential applications [35,38,40,42]. Importantly, the presence of promising toxin candidates as novel therapeutics or biopesticides is not correlated with their harmful effects on humans. Thus, even venomous species that are completely harmless to humans (which are, by far, the majority of all venomous organisms) might contain potentially interesting toxins in their venoms that could benefit humanity. In addition, the rapid technical advancement of modern -omics techniques has increased not only the speed of venom research but also the depth and quality of generated data and enabled a deeper understanding of various aspects relating to venom evolution and biochemistry. Furthermore, improvements in the sensitivity of modern research equipment have enabled access to venoms from much smaller specimens, such as tiny pseudoscorpions of only a few millimetres in length [53,54]. With the vast majority of venomous animals being less than 1 cm in size, a continuous improvement in the sensitivity of equipment and assays will further increase the quantity and diversity of venomous animals that are accessible to future research. The diversity of venom components is further increased by some animals producing specific venoms for different purposes [55,56,57] and the potential of microorganisms living inside the venom glands, which also contribute to the chemical complexity of animal venoms [58].

## 5. Contributions to This Special Issue

This Special Issue of *Biomedicines* comprises 12 research and four review articles about a wide range of venomous or poisonous invertebrates and vertebrates, including sea anemone, cone snails, leeches, spiders, scorpions, ants, caterpillars, frogs, and snakes. The breadth of these contributions covers not only their taxonomic diversity but also both the detrimental and beneficial aspects of animal toxins from a human perspective, which is reflected in the title of this Special Issue: “Animal Venoms—Curse or Cure?”. The “curse” aspect of venoms is covered in a number of contributions. For example, the review articles by Ahmadi et al. [59] and Seldeslachts et al. [60] discuss the dangers that venomous scorpions and caterpillars pose to humans, but they also provide insights into current and future treatment options, such as the next-generation recombinant antivenoms [59]. Two contributions from Jeroen Kool’s group examine the usefulness of the small-molecule PLA2 inhibitor Varespladib as a potential drug for the treatment of snake bites. In these studies, cutting-edge nanofractionation analytics are employed to determine the effects of Varespladib and other small molecules on the coagulopathic effects of various crotalid and viperid snake venoms [61,62]. The Hodgson lab examined the neutralising abilities of different antivenoms against the effects of venom from the Chinese cobra by using the chick biventer nerve muscle preparation [63]. Another study on Australian snake venoms by Isbister et al. found that phospholipase A2 levels in human snakebite victims could be used as an early indicator of envenomation by Australian elapids (with exception of brown snakes) [64]. The contribution by Nixon et al. reveals that the dimeric ant peptide Mp1a is responsible for a broad range of activities, including the extremely painful symptoms experienced by humans that are stung by jack jumper ants [65]. Potential biosecurity concerns of conotoxins are discussed and largely rejected by Bjorn-Yoshimoto et al. [66], with the benefits of peptides from cone snails by far outweighing their potential negative impacts. Nevertheless, this review article nicely exemplifies how scientifically unsubstantiated political red tape can negatively impact the progress of toxinological research.

Several other contributions to this Special Issue cover the “cure” aspect of animal venoms, which is their potential usage for the benefit of humans. The review by Lemke and Vilcinskas, for example, highlights the resurging interest in leeches, which have been used in traditional medicine for thousands of years [14]. Today’s research focusses on a variety of bioactive leech peptides and proteins affecting blood coagulation and inflammation. Spider venom peptides, on the other hand, might be promising candidates for novel analgesics by targeting particular subtypes of voltage-gated sodium (Na_V_) channels. Yin et al. [67] not only provide the first characterisation of a toxin from the theraphosid genus *Poecilotheria*. They also demonstrate that, by simply introducing one additional residue, the toxin is converted from a Na_V_1.7 activator into an inhibitor, which can be crucial for designing effective analgesic leads. Novel toxins with potential anti-inflammatory activity are further reported from the venom of a sea anemone [68]. In addition to their proposed medical applications, venom components are also useful tools for research. The structural diversity of peptide toxins from venoms, for example, provides an excellent source of novel modulators for studying the pharmacological properties of ion channels and receptors. The article from Wilson et al. [69] describes the new α-conotoxin Pl168 from *Conus planorbis*, belonging to the well-known A superfamily of conus toxins. However, unlike other members of the A superfamily, Pl168 shows no activity on a range of nAChRs or Ca^2+^ and Na^+^ channels. Pl168 also comprises a new structural type within the A superfamily, with a presumed novel pharmacological target. Even more potential pharmacological probes could be hidden among the peptides [70] and small molecules [71] found in scorpion venoms. Another study adds to the list of venom compounds with interesting pharmacology by employing a combined transcriptomic and proteomic approach to uncover not only some new bradykinin potentiating peptides but also the first evidence of three-finger toxins from a viperid snake venom [72]. On the other hand, a bradykinin-antagonising peptide was identified from a Chinese frog species and characterised by Zhou et al. [73].

## 6. Conclusions

I trust that the audience of this Special Issue of *Biomedicines* will enjoy reading the excellent contributions from many of the leading researchers in the field. I further hope that they will inspire the next generation of scientists to turn their attention towards studying the fascinating world of animal venoms and toxins. Despite the horrible and yet too often fatal consequences that venomous animals (in particular, snakes) can have on humans, I believe that their potential benefits for basic research and towards health and food production for billions of people by far outweigh their negative effects. Continuous progress in antivenom development will help in making antivenoms more efficient, cheaper to produce, and more tolerable by reducing unwanted side effects. Thus, continued toxinological research will help in further reducing the negative effects of animal venoms, thereby tipping the scales even more in favour of their beneficial effects for humanity.

## Data Availability

Not applicable.
